# Coding potential of the products of alternative splicing in human

**DOI:** 10.1186/gb-2011-12-1-r9

**Published:** 2011-01-20

**Authors:** Guido Leoni, Loredana Le Pera, Fabrizio Ferrè, Domenico Raimondo, Anna Tramontano

**Affiliations:** 1 Dipartimento di Scienze Biochimiche, Sapienza Università di Roma, P.le A. Moro, 5 - 00185 Rome, Italy; 2 INRAN, Via Ardeatina, 546 - 00178 Roma, Italy; 3 Istituto Pasteur Fondazione Cenci Bolognetti, Sapienza Università di Roma, P.le A. Moro, 5 - 00185 Rome, Italy

## Abstract

**Background:**

Analysis of the human genome has revealed that as much as an order of magnitude more of the genomic sequence is transcribed than accounted for by the predicted and characterized genes. A number of these transcripts are alternatively spliced forms of known protein coding genes; however, it is becoming clear that many of them do not necessarily correspond to a functional protein.

**Results:**

In this study we analyze alternative splicing isoforms of human gene products that are unambiguously identified by mass spectrometry and compare their properties with those of isoforms of the same genes for which no peptide was found in publicly available mass spectrometry datasets. We analyze them in detail for the presence of uninterrupted functional domains, active sites as well as the plausibility of their predicted structure. We report how well each of these strategies and their combination can correctly identify translated isoforms and derive a lower limit for their specificity, that is, their ability to correctly identify non-translated products.

**Conclusions:**

The most effective strategy for correctly identifying translated products relies on the conservation of active sites, but it can only be applied to a small fraction of isoforms, while a reasonably high coverage, sensitivity and specificity can be achieved by analyzing the presence of non-truncated functional domains. Combining the latter with an assessment of the plausibility of the modeled structure of the isoform increases both coverage and specificity with a moderate cost in terms of sensitivity.

## Background

Alternative splicing (AS) is a mechanism used by cells to diversify the proteins produced by a gene. Estimates of the amount of AS in human have risen dramatically over recent years, especially since the advent of novel high-throughput sequencing technologies [1-3], reaching up to the 95% of the multi-exon genes [4].

While the role of AS in expanding the functional complexity of a genome is established, less clear is whether all generated transcripts do indeed encode functional proteins and therefore expand the coding potential of a genome. Cases are known of events that produce splicing variants (isoforms) showing novel and sometimes unexpected structural and functional properties [5,6]. On the other hand, evidence from analysis of sequences, structures and homology models suggest that many AS isoforms, even if detectable at the transcriptomic level, might not encode functional proteins because, for example, they lack important functional regions and/or seem to correspond to incomplete structures [7,8].

The overwhelming majority of AS evidence is based on transcriptomic data; therefore, a proof that the splicing product is eventually translated and can fold into a functional protein is generally missing. Nonetheless, it is evident that knowing whether or not an isoform observed at the transcriptional level does indeed correspond to a functional protein is relevant for both theoretical and practical reasons. Since it is practically impossible to identify negative cases - examples where one isoform certainly does not correspond to a functional protein - this is a scenario where we can only resort to computational methods for obtaining a probabilistic estimate of the likelihood that a protein is functional.

Computational method inferences are difficult to validate in the absence of a clearly defined negative set, but one can still assess their sensitivity in identifying isoforms that are known to be translated because, for example, they have been unambiguously identified in proteomic experiments. Although the detection of a peptide identifying an isoform is not conclusive for its functional characterization, it does imply that the corresponding transcript is translated into a protein likely to fold and be produced at sufficient levels to be detected, and therefore strongly suggests that it is unlikely to be non-protein coding. This concept has been applied in the past, in small scale, to data by Tanner and coworkers [9], who found 16 human genes for which two different isoforms could be unambiguously identified by mass spectrometry (MS). A larger scale systematic analysis of isoform proteomic identification based on MS data performed for the fruit fly [10] led to the identification of AS events that could be confirmed at the protein level for 130 genes. The limited coverage of proteomics data, still far from the level of completeness provided by transcript expression analysis platforms [10], is the main reason behind the relatively low number of genes identified in both the aforementioned studies.

In this work, we take advantage of MS data for constructing a dataset composed of human isoforms unambiguously identified by MS (AS positive (ASPos) dataset) and use several computational methods to compare their properties with those of isoforms for which no matching peptide can be found in MS public database (unknown dataset). In particular, we study: their structural plausibility, based on structural models by homology; the presence of complete domains, based on Pfam domain definitions [11]; and the presence of functional sites, such as catalytic sites, based on SwissProt annotated features [12].

The results obtained with this positive dataset, which we used as a benchmark, allowed us to estimate how much each of the methodologies listed above can help in identifying translated isoforms. There is clearly a trade-off between the coverage achieved by each method (for example, the presence of a functional domain is more frequent than the presence of annotated functional sites) and their reliability in predicting the likelihood that the isoform is translated into a product. We used our positive set to estimate the fraction of false negatives detected by each method separately and by their combinations. In order to validate our conclusions, we also built two additional datasets, one containing ORFs obtained from the translation of non-coding transcripts (negative dataset) and one including all products of genes not undergoing AS and for which experimental evidence is available by MS (the noASPos dataset). The first dataset is somewhat artificial since its elements are only selected on the basis of the absence of termination codons in a sufficiently long ORF (at least 100 amino acids long) and is not really representative of realistic cases. On the other hand, the unknown dataset might contain isoforms that are not observed because they are only present at specific times or in specific cell types and isoforms that are not detected for technical reason by MS. This notwithstanding, the analysis of both datasets can be used to obtain an estimate of the false positive rate of the computational techniques.

Results obtained by considering the ASPos and unknown datasets show that, as expected, the single method with highest sensitivity - that is, the ability to correctly identify translated products - can be achieved by relying on the conservation of features annotated in SwissProt in the isoform, but this is not very frequent (coverage of about 14%), while a reasonably high coverage (81%), a good sensitivity (95%) and a specificity above 40% can be achieved by analyzing the presence of non-truncated Pfam domains. Combining the latter with an assessment of the plausibility of the modeled structure of the isoform increases the coverage by another 8%, with a decrease in sensitivity, but in this case the lower estimate for the specificity increases by at least 2%.

When the artificial non-coding dataset is used as a negative set, the results do not change substantially. Clearly, no SwissProt annotations exist for these transcripts, the presence of non-truncated Pfam domains still has the highest coverage (81%) and sensitivity (95%), with a specificity of around 30%. Also, the combination of structural plausibility and the presence of non-truncated Pfam domains produces a similar picture with coverage, sensitivity and specificity values of 87%, 93% and 33%, respectively.

A different balance between specificity and sensitivity can be required in different cases; therefore, we think that the results reported here can provide a useful guide to prioritizing experiments for different purposes.

## Results and discussion

Proteomics technology can provide experimental evidence that a specific isoform is expressed, translated and sufficiently stable to be detected *in vivo*, although, unfortunately, it cannot be used to exclude the presence of a protein, nor can any other experimental technique provide such information. Nevertheless, the analysis of proteomic datasets can offer a repertoire of isoforms whose products are certainly present in the cell. Proteomics experiments provide a large amount of data, which are available in specialized databases such as PeptideAtlas [13] in the form of peptides with unambiguous mapping to protein sequences. In order to be able to detect the presence of a specific isoform in the midst of the whole spectrum of possible products of a gene, we first need to identify those isoform regions that are specific for one isoform, that is, that do not map to any other isoform of the same gene [14].

**Figure 1:**
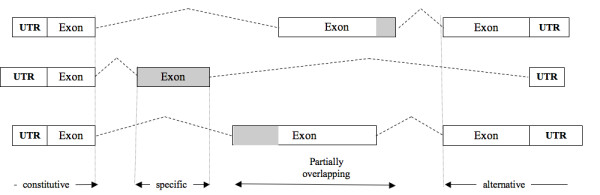
**Scheme of possible scenarios for comparing different isoforms**. Only peptides mapping in the products of shaded regions are considered specific.

Of the 22,320 Ensembl57 [15] protein coding genes, 15,914 produce more than one isoform, and are therefore subject to AS. We did not include in this dataset those isoforms annotated as non-protein coding by Ensembl, and those differing only in their UTRs at the 5' or 3' end (therefore having identical coding regions), and ended up with 60,568 isoforms. In this group of alternative transcripts, we identified all regions (whole exons or exon portions) of each gene that are included in only one isoform (Figure 1). The detection of peptides mapping to such specific regions in MS experiments allows the unambiguous identification of the translation of the corresponding transcripts. PeptideAtlas human build peptides (May 2010) were mapped to the exons of these isoforms and classified as specific or unspecific accordingly. A total of 1,124 isoforms (from 1,025 genes) are identified by at least one specific peptide, and represent the set of isoforms whose existence is confirmed at the protein level. This figure is somewhat different from that reported in [14], where specific transcripts for 3,059 human alternatively spliced genes were identified using PeptideAtlas peptides, but this was expected since we used a more up-to-date release of PeptideAtlas in which the peptide mapping criteria were more stringent.

We focused our analysis on those genes having at least one isoform unequivocally identified by PeptideAtlas peptides and at least one other isoform for which no peptide mapping to its specific regions was found and that were predicted by PeptideSieve [16] to be detectable by the most popular current MS technologies (this being due to their charge, hydrophobicity, mass, secondary structure, and so on). When more than one ASPos or unknown isoform were present for a gene, we selected only the shortest and longest ones, respectively. We also verified that the results would not be affected if we were to use isoforms identified by at least two or more peptides (data not shown).

We also built a dataset containing the products of all genes that do not undergo AS and that are identified by at least one peptide present in PeptideAtlas and a dataset built by translating ORFs present in processed transcripts annotated as non-coding in Ensembl (see Materials and methods).

In conclusion, our datasets include 555 isoforms identified by MS (ASPos dataset), 555 isoforms corresponding to the same genes but for which no specific peptide is present in PeptideAtlas (unknown dataset), 865 products of genes that do not undergo AS (noASPos dataset) and 555 translated sequences from non-coding transcripts (negative dataset).

Our unknown dataset doubtlessly includes isoforms whose product is not detected since it is present only in specific tissues, cell cycle phases, developmental stages, or in the presence of specific stimuli, but a certain fraction can produce non-protein coding transcripts.

Our aim is to determine how many of the isoforms in the ASPos dataset that are identified as true products of a regulated AS event can be detected by different computational methods in order to evaluate their sensitivity. For the reason described above, the unknown set can only be used to estimate the lower limit of the specificity of the methods, while the negative dataset does not suffer this problem but is less representative of a real situation, even though we obtained the sequence by translating processed transcripts rather than random genomic sequences.

### Positive isoforms are predicted to be structurally more plausible than unknown isoforms

Arguably, a considerable amount of non-functional AS will lead to polypeptide sequences that can not fold in a stable conformation and therefore are quickly degraded. We cannot exclude that a stable conformation can be the result of profound structural rearrangements or of the establishment of stabilizing interactions with a binding partner. Such cases are very hard to identify, but apparently not very frequent [5,6].

Here we predicted the structure of all the isoforms of the ASPos and noASPos datasets for which a suitable structural template could be found and carefully analyzed the resulting models according to several criteria. We estimated how well packed the protein model is and the extent to which hydrophobic surface is exposed to solvent with respect to the average single domain proteins in the database of solved protein structures and to the template used to build the model (see Materials and methods). We also assessed whether insertions and deletions with respect to the structural template, when present, could be accommodated within the modeled structure. In particular, we flagged as 'unlikely' cases where a deletion would imply connectivity between two residues that are too far away in space and where insertions would occur in the well packed core of the protein.

The detailed pipeline for model building is described in the Materials and methods section. We were able to model 230 isoforms from the noASPos dataset, 147 from the ASPos dataset, 145 from the unknown dataset and 84 from the negative dataset, with coverage (that is, the fraction of protein sequence that can be modeled) of at least 90%.

The majority (134; 91%) of modeled isoforms from the ASPos dataset are structurally consistent. Difficult to accommodate deletions and/or insertions with respect to the template are present for nine isoforms from the positive dataset (6%), while five show a non-optimal packing of their interior (the two cases can obviously occur in the same isoform). The corresponding numbers for the noASPos dataset are similar (88% with a plausible structure, 9 isoforms with difficult to accommodate deletions/insertions corresponding to about 4% of the total and 18 with non-optimal packing corresponding to 8% of the total). On the other hand, the fraction of viable models is remarkably smaller for the negative (40; 48%) and unknown dataset isoforms (69; 48%). The negative dataset includes 13 models with difficult to accommodate deletions/insertions and 32 models with non-optimal packing. In 64 cases the models of the unknown dataset show non-optimal packing and in 10 they also have difficult to accommodate deletions/insertions.

### Functional domains are more often truncated in unknown isoforms than in positive ones

AS can remove whole protein domains, but tend not to occur within domains [17]. While this is not an absolute rule, it is reasonable to assume that a substantial amount of isoforms where at least one domain is truncated by a splicing event correspond to non-protein coding transcripts.

To verify how well this criterion performs in real cases, we used the definition of domains in the Pfam database [11]. Each Pfam domain is described by a hidden Markov model (HMM) built on the seed example sequences for that domain. Different isoforms of the same gene can carry different sets of domains [18,19]. On the other hand, a domain that is truncated in an isoform is more likely to be the result of incorrect splicing than of a regulated event. As described in Materials and methods, a domain is considered truncated if the isoform sequence matches less than 70% of its length.

Most isoforms of the noASPos and ASPos datasets (83% and 86%, respectively) only include complete Pfam domains, and only 5% contain truncated Pfam domains. The situation is drastically different for the unknown and negative datasets, where 41% and 50% of the isoforms only contain complete Pfam domains, respectively, and 42% and 36% include at least one truncated Pfam domain.

It should be mentioned that more Pfam domains are found in isoforms of the ASPos dataset than the unknown one (2.43 domains on average versus 1.63). This could be attributed to the fact that these isoforms tend to be longer than the unknown ones (average length 694 amino acids versus 345). On the other hand, our data indicate that the length has little or no impact on the number of truncated domains. The average length of proteins included in the PDB database [20] is 606 amino acids and the percentage of proteins with truncated Pfam domains is only 14%, much lower than what we observe in our unknown dataset, and the percentage of truncated Pfam domains in these proteins is independent of the length (data not shown). Similarly, sequences in our noASPos dataset, whose average length (436) is comparable with that of members of the unknown (345) and negative datasets (485), include a truncated domain in only 5% of cases (compared with 42% and 36% for the unknown and negative datasets). Although we cannot exclude that the length of the transcripts might affect the results to a minor extent, we believe that in any case, the presence of a truncated domain, whatever the reason, is an indication of a lack or impairment of the associated function.

### Functional features are rarely disrupted in positive isoforms

We verified whether AS would remove existing annotated active sites present in other isoforms of the same gene in the ASPos and unknown datasets. A similar procedure cannot be applied to the negative dataset, since these translated sequences are not annotated, nor, obviously, to the noASPos dataset, where no AS occurs.

In several entries of our ASPos dataset, the annotation for an active site (80 genes) is present in the Swiss-Prot database [12]. Active sites are present in both isoforms of the positive and unknown dataset in only 60% of cases. In all other cases, the isoform of the unknown dataset (which is not identified by a specific peptide in PeptideAtlas) does not retain the active sites. In a single case a functional site is found in the unknown dataset isoform (Ensembl ID: ENSP00000359932) but not in the associated positive isoform (ENSP00000359935). In this case, positive and unknown isoforms have a radically different amino acid sequence after residue 114, due to the usage of different exons after the initial shared portion (for this reason these two isoforms are associated with different SwissProt IDs, FPGT_HUMAN and TNI3K_HUMAN, respectively) and have a different biological function (the former is a fucose-1-phosphate guanylyltransferase, the latter a serine/threonine-protein kinase). Therefore, in this particular case, the loss of functional sites is due to a radical functional change.

### Transcription levels of the isoforms in different datasets

**Figure 2:**
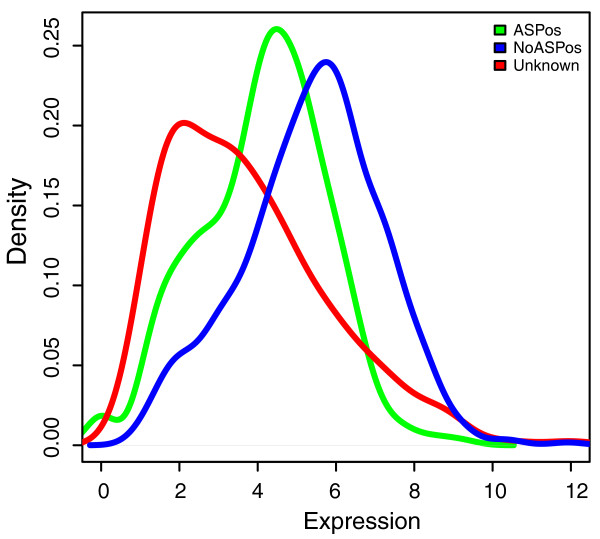
**Distribution of expression level values for specific exons of transcripts included in the noASPos, ASPos and unknown datasets**.

Isoform-specific expression can be estimated by means of recently developed microarray platforms that target all exons of a gene or (a subset of) exon-exon junctions. The Affymetrix Exon arrays [21] are high-density chips in which probe sets (composed by at least four probes) were designed for all exons in Ensembl. This platform proved to be very accurate and sensitive in the detection of AS events and is routinely used for the study of cellular processes in healthy or disease conditions. Expression data from 11 adult human tissues are publicly available from the Affymetrix website, and offer a valuable resource for the study of exon-level expression of human isoforms. The distribution of the expression level of the transcripts present in the ASPos, noASPos and unknown datasets are shown in Figure 2.

As can be appreciated from Figure 2, the isoforms in the unknown dataset have a lower level of normalized expression of specific exons than those in the ASPos and noASPos datasets. This notwithstanding, the overlap between the distributions is rather high (around 70%). Some of the unknown isoforms whose transcripts are expressed at lower levels might correspond to products present in limited amounts and less likely to be detected by MS, or they might be due to splicing errors, which are expected to happen at low frequency. On the other hand, a high percentage of non-detected isoforms are expressed at levels similar to those of the positive datasets and the lack of their detection points to the possibility that they do not produce functional proteins.

### Statistical significance of different criteria

**Figure 3:**
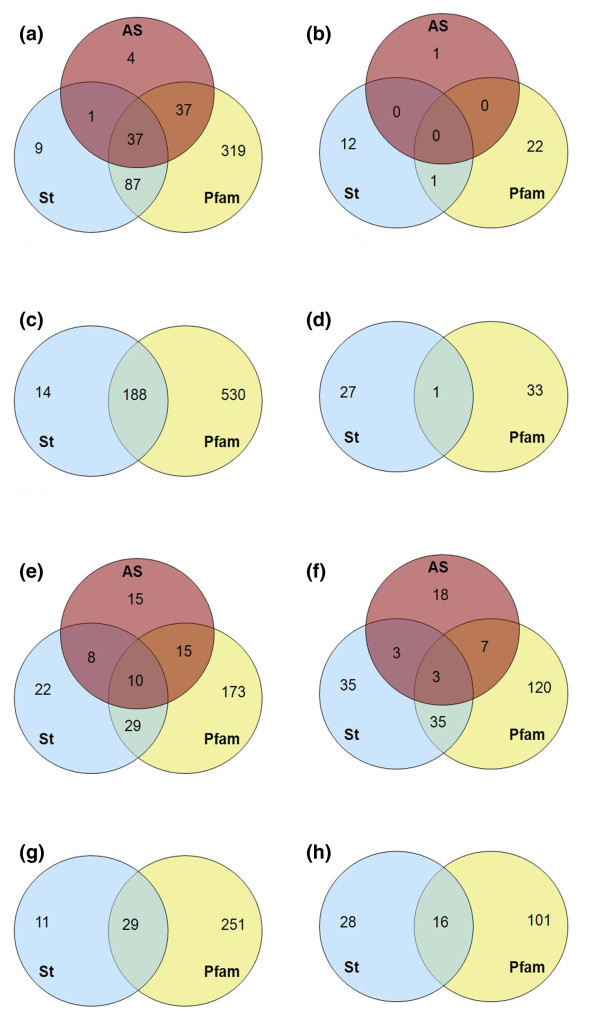
**Venn diagrams showing the number of isoforms predicted to be functional and unlikely to be functional according to each method**. (a-h) The number of isoforms predicted to be functional according to each method in the ASPos dataset (a), the noASPos dataset (c), the unknown dataset (e) and the negative dataset (g) and the number of isoforms unlikely to be functional according to each method in the ASPos dataset (b), the noASPos dataset (d), the unknown dataset (f) and the negative dataset (h). AS, preservation of active sites; Pfam, completeness of Pfam domains; St, structural plausibility.

Figure 3 and Table 1 summarize results on coverage, accuracy, sensitivity and the lower estimate of the specificity of the criteria described above as well as of their union and intersection.

**Table 1:** Results of the statistical analysis with respect to the unknown dataset

	Coverage	TP	TN	FP	FN	Accuracy	Sensitivity	Specificity
AS	0.14	79	31	48	1	0.69	0.99	0.39
St	0.26	134	76	69	13	0.72	0.91	0.52
Pfam	0.81	480	165	227	23	0.72	0.95	0.41
AS U St	0.35	175	101	99	14	0.71	0.93	0.50
St U Pfam	0.89	490	203	257	35	0.71	0.93	0.44
AS U Pfam	0.85	485	186	250	24	0.71	0.95	0.43
AS U St U Pfam	0.92	494	221	272	36	0.70	0.93	0.45
AS ∩ St	0.06	38	6	18	0	0.71	1.00	0.25
St ∩ Pfam	0.18	124	38	39	1	0.80	0.99	0.49
AS ∩ Pfam	0.10	74	10	25	0	0.77	1.00	0.28
AS ∩ St ∩ Pfam	0.05	37	3	10	0	0.80	1.00	0.23

Coverage, accuracy, sensitivity and specificity of the different strategies and their combinations (U = union and ∩ = intersection) with respect to the unknown dataset. AS, preservation of active sites; Pfam, completeness of Pfam domains; St, structural plausibility. The definition of the other parameters is reported in Materials and methods. FN, false negative; FP, false positive; TN, true negative; TP, true positive.

There is an obvious trade-off between coverage and accuracy. Isoforms that preserve the active sites, that contain only non-truncated Pfam domains and that are structurally plausible are very likely to be translated in functional products (80% accuracy), although this combination of features is only observed in a small fraction of the cases. On the other hand, a very good compromise for predicting the functionality of an isoform is to verify that it does not contain interrupted Pfam domains or has a plausible modeled structure. This would be appropriate for most practical purposes and applicable to almost 90% of the cases, providing an accuracy above 70% with a sensitivity and specificity of 93% and of at least 44%, respectively. The detection of complete Pfam domains, certainly easier to obtain in large scale analyses, has a high coverage and good sensitivity, although its specificity is not very high. In practice, when an isoform contains an interrupted Pfam domain, it is very likely not to be functional, while the detection of only complete Pfam domains in an isoform, especially if produced by a transcript overlapping with a coding one, as is the case for our non-coding dataset, is not very informative. The overall picture does not change when the non-coding dataset is considered instead of the unknown one (in which case, however, the conservation of the active sites cannot be taken into account), as shown in Table 2.

**Table 2:** Results of the statistical analysis with respect to the negative dataset

	Coverage	TP	TN	FP	FN	Accuracy	Sensitivity	Specificity
St	0.21	134	44	40	13	0.77	0.91	0.52
Pfam	0.81	480	117	280	23	0.67	0.95	0.29
St U Pfam	0.87	490	145	291	35	0.66	0.93	0.33
St ∩ Pfam	0.15	124	16	29	1	0.82	0.99	0.36

Coverage, accuracy, sensitivity and specificity of the different strategies and their combinations (U = union and ∩ = intersection) with respect to the negative dataset. Pfam, completeness of Pfam domains; St, structural plausibility. The definition of the other parameters is reported in Materials and methods. FN, false negative; FP, false positive; TN, true negative; TP, true positive.

## Conclusions

The wealth of high throughput data that are continuously being produced opens the way to the investigation of relevant properties of living organisms and can be effectively exploited in many instances. Cataloguing all putative isoforms of genes is one such example, although care should be taken since there is evidence that not all isoforms identified at the transcriptional level correspond to functional proteins [8].

The question that we address here - whether or not an isoform is likely to be functional - is relevant but unfortunately cannot be answered in a definite way by experimental approaches. While the presence of a functional protein in the cell can be demonstrated, it is impossible to assess that a given peptide sequence is not present or functional at any given time or in any compartment of a cell or an organism. Computational methods, provided they are properly assessed and evaluated, are therefore essential. We show here that different computational strategies and their combination can be effectively used as proxies for assessing the likelihood that an isoform observed at the transcriptional level does correspond to a functional protein product.

We believe that the estimate of the accuracy of different computational strategies and of their different combination provided here can be used for selecting different strategies for different occasions. In some cases, a higher rate of false positives might be preferable to a higher number of false negatives - for example, when a specific gene of interest is being investigated thoroughly - although even in this case prioritizing the experiment taking advantage of computational estimates can save time and resources.

Obviously, the impossibility of obtaining a true negative set implies that, while one can assess the ability of the methods to detect translated isoforms - that is, the percent of true positives and false negatives that they predict - it is impossible at present to give a precise estimate of how many false positives would result from any computational analysis. This is a very difficult, or perhaps impossible, problem to solve, but learning about the ability of the analyzed strategies to detect most of the truly translated isoforms and the lower estimate of their specificity that we have provided here can be of great help in understanding the functional repertoire of higher eukaryote genomes. Clearly, the accumulation of more and more proteomic data will allow even more effective strategies to be devised.

## Materials and methods

The datasets used in this analysis were all constructed starting from the coding portion of the human genome in Ensembl57 [15]. Out of the total number of Ensembl protein coding genes (22,320), 6,406 genes are not subjected to AS. Of all the isoforms encoded by the remaining genes, 31,618 are classified as non-protein coding according to the Ensembl annotations. In the remaining genes, we found 7,467 isoforms differing only for their untranslated regulatory regions from other isoforms in the same gene, and these were removed. We also discarded an additional 1,844 genes that were left with only one isoform. At this stage the dataset of alternative spliced isoforms contains 60,568 isoforms encoded by 13,980 genes.

Taken together, these latter isoforms contain 278,155 exons in their coding sequences identified by a unique Ensembl exon ID. These exons were classified as present in all transcripts of a gene (constitutive, 20% of the total), in a subset of the gene transcripts (semi-constitutive, 49%), or in a single transcript only (specific, 12%). Cases of semi-constitutive exons with parts of their sequence partially overlapping with another exon were classified in a separated category (partially overlapping, 19%).

### Mapping of proteomic peptides on the human AS isoforms

Proteomics data were retrieved from the PeptideAtlas database [13]. PeptideAtlas organizes its data into builds centered on a particular species or tissue. We used the May 2010 human build [22], which contains 71,303 different peptides ranging in size from 7 to 66 (mean 17); these were unambiguously mapped to human Ensembl57 proteins. We selected only those peptides classified by PeptideAtlas as non-exon spanning. Of these, 39,956 match isoforms included in our dataset. We classified 11,005 peptides that unambiguously identify one protein isoform by mapping to a specific exon or to a specific part of partially overlapping exons as 'specific' peptides. Peptides that map into semi-constitutive exons, constitutive exons or non-specific parts of partially overlapping exons were classified as 'unspecific' peptides.

### Building of the positive, negative and unknown datasets

The noAS positive dataset was built by selecting the products of all non-alternatively spliced genes that are unambiguously identified by PeptideAtlas peptides and contains 865 gene products identified by 4,589 peptides. All the isoforms produced by AS that are unambiguously identified by specific PeptideAtlas peptides (576 isoforms identified by 2,546 peptides) were considered for inclusion in the ASPos dataset. Out of all remaining isoforms, those having specific exons (or specific exon regions in partially overlapping exons) but that were not identified by any PeptideAtlas peptide, although they could in principle be detected according to the PeptideSieve algorithm [16], were considered for inclusion in the unknown dataset (782 isoforms). In detail, the sequences of the isoforms in the unknown dataset were submitted to the PeptideSieve algorithm, which predicts the likelihood of the peptide being observed in a proteomics experiment, taking into account ionization and missed cleavage propensity. The program first performs an *in silico *digestion of the protein and then computes for each peptide a list of physical and chemical descriptors. Next, it scores the likelihood that each peptide is observed in one of the four proteomics platforms (PAGE MALDI, PAGE ESI, ICAT ESI, MUDPIT ESI). An unknown isoform is considered detectable in a proteomics experiment if at least one of its peptides, originating from its specific regions, has a score of at least 0.5 (the default lower limit score in PeptideSieve). When used as described above, PeptideSieve has an expected accuracy above 85%. When more than one identified or not identified isoform was present in the same gene, we included only the shortest one of the ASPos dataset and the longest one of the unknown dataset. At the end of the procedure the ASPos and unknown datasets included 555 isoforms each.

To obtain the negative dataset, we considered transcripts annotated as non-coding present in regions of the genome containing at least one coding transcript (we did not include transcripts undergoing nonsense mediated decay), translated their sequence starting from the first AUG and continuing until a stop codon was encountered, and selected the longest 555 translated sequences. The average lengths of members of the datasets are 436 (noASPos), 694 (ASPos), 345 (unknown) and 485 (negative).

### Structural characterization of the isoform datasets

We built structural models by homology for each isoform in our datasets for which the native structure is unknown, and for which a suitable template covering more than 90% of the sequence could be found. HHsearch 1.1.5 [23] was used to search for possible structural templates (default parameters) and for obtaining the sequence alignment between the target and its putative templates. The resource builds a HMM of the target protein family and compares it to the HMMs representing a set of non-redundant families of proteins of known structure (sequence identity between any pair below 70%). Model building was performed using a local version of Modeller9v8 [24] (default parameters). Models were considered structurally plausible if there is a deletion with respect to the template and the distance between the two residues on either side is larger than 15Å; if there is an insertion of more than three residues in the core of the protein, that is, between two residues whose solvent accessibility calculated with POPS [25] is lower than 5Å^2^; if the packing efficiency of the resulting model computed using the OS software [26] is below 0.54 while that of the template used for modeling is not; and if the 'packing-eff' computed using the NUCPROT package [27] is below 25.9, while that of the template used for modeling is not. The thresholds for POPS, Packing-eff and OS tools were derived by running the programs on 4,122 monomeric proteins solved by X-ray crystallography at a resolution better than 2Å. The chosen thresholds, 25.5 for POPS values, 25.9 for Packing-eff values and 0.54 for OS values, correspond to two standard deviations from the average (data not shown).

### Functional domain characterization of the isoform datasets

We mapped Pfam domains [11] on the protein sequences of the isoforms in the datasets, using the batch search utility available through the Pfam web interface, using Pfam-A families and an E-value below 10E-5. For each domain, we computed the coverage of the HMM representing the domain: all domains assigned to an isoform whose length covers less than 70% of the corresponding HMM were considered truncated. This threshold was chosen by evaluating the HMM coverage in a set of protein sequences for which the structure is known. We extracted 3,859 monomeric structures with less than 30% sequence identity from PDB [20]. For every protein, the corresponding Uniprot sequence was retrieved and Pfam domains were assigned according to the criteria described above; 93% of Pfam domains have a coverage between 0.70 and 1.0 in these sequences (data not shown).

### Mapping of Swiss-Prot features on the isoform datasets

Active site residues as annotated in Swiss-Prot (release 57, March 2009) were mapped on all isoforms encoded by a gene using the Ensembl *perl *APIs and using in-house developed tools.

### Evaluation of transcriptomic expression

Exon-level isoform expression was extracted from Affymetrix Exon 1.0 ST Array public datasets [28]. The profiled human tissues include breast, cerebellum, heart, kidney, liver, muscle, pancreas, prostate, spleen, testis, and thyroid. RNA-normalized probe-set expression levels, computed using the Affymetrix Power Tools (APT), are available from the Affymetrix web site. We were able to retrieve isoform-specific expression levels for 728 and 532 isoforms of the noASPos and ASPos datasets, respectively, and 264 isoforms of the unknown dataset. Probe set expression levels were computed as the median of normalized expression values in the 11 tissues in the panel. Isoform expression is estimated as the median expression of all probe sets falling in the isoform-specific regions.

### Data analysis

We used the following definitions: true positive (TP) - an isoform in the positive dataset for which the considered descriptor is consistent with the hypothesis of the isoform being functional (that is, structurally plausible, or not containing truncated domains, or containing an active or binding site); false negative (FN) - a positive set isoform for which a descriptor suggests loss of functionality (that is, structurally not plausible, or containing a truncated domain, or missing active sites present in some other isoform of the same gene); false positive (FP) - an unknown or negative set isoform for which the considered descriptor is consistent with the hypothesis of the isoform being functional; true negative (TN) - an unknown or negative set isoform for which a descriptor suggests loss of functionality. We can use the confusion matrix originated by the values of TP, FN, FP, TN to evaluate how well each descriptor (and all their intersections and unions) is able to discriminate between isoforms in the datasets, using the commonly used measures accuracy (ratio between correct predictions TP + TN and total predictions TP + FN + FP + TN), sensitivity (ratio between correct positive predictions TP and total predictions in the positive dataset TP + FN), and specificity (ratio between correct negative predictions TN and total predictions in the negative or unknown dataset TN + FP). Since each descriptor can be applied only to a subset of the total isoforms (for example, not all isoforms can be modeled, not all isoforms have Pfam domains, and not all isoforms have an annotated active site), the coverage of each descriptor (defined as the number of isoforms to which the descriptor can be applied over the total number of isoforms under examination) is highly variable. The union of two descriptors, or of all three of them, obviously increases the coverage at the cost, in some cases, of accuracy. When considering the union of two or three descriptors, one must take into account that a number of isoforms can have discordant descriptors. For example, a given isoform of the positive dataset can be structurally plausible, thus having one truncated domain, and therefore it is a TP from the point of view of the structural descriptor ad a FN for the domain integrity descriptor. In all these cases we counted these isoforms as FN (or as TN for isoforms belonging to the negative or unknown dataset).

## Abbreviations

AS: alternative splicing; ASPos: alternatively spliced positives; FN: false negative; FP: false positive; HMM: hidden Markov model; MS: mass spectrometry; noASPos: non alternatively spliced positives; ORF: open reading frame; TN: true negative; TP: true positive; UTR: untranslated region.

## Competing interests

The authors declare that they have no competing interests.

## Authors' contributions

GL, LL, FF and RD carried out the analysis and helped to draft the manuscript. AT conceived of the study, participated in its design and coordination and helped to draft the manuscript. All authors read and approved the final manuscript.
